# Association between canakinumab dose and long-term remission in Still’s disease: insights from the AIDA network registry

**DOI:** 10.3389/fphar.2026.1846937

**Published:** 2026-08-10

**Authors:** Antonio Vitale, Valeria Caggiano, Jessica Sbalchiero, Giuseppe Lopalco, Abdurrahman Tufan, Gaafar Ragab, Petros P. Sfikakis, Lorenzo Dagna, Ezgi Deniz Batu, Seza Ozen, Fabrizio Conti, Federica Maiolini, Serena Bugatti, Micol Frassi, Piero Ruscitti, Maria Morrone, Francesco La Torre, Ibrahim Yahya Cakir, Nergis Akay, Elif Kilic Konte, Katerina Laskari, Yelda Bilginer, Angelica Gattamelata, Jacopo Croce, Ludovico De Stefano, Giulia Voltarel, Paola Cipriani, Maria Cristina Maggio, Sulaiman M. Al-Mayouf, Lampros Fotis, Donato Rigante, Jurgen Sota, Elena Verrecchia, Giuliana Guggino, Lidia La Barbera, Matteo Piga, Giacomo Emmi, Romina Gallizzi, Giovanni Conti, Paolo Sfriso, Elena Bartoloni, Roberto Giacomelli, Patrizia Barone, Alma Nunzia Olivieri, Annachiara Alemanno, Alberto Lo Gullo, Haner Direskeneli, Fatma Alibaz-Oner, Anastasios Karamanakos, Marcella Prete, Amr Edrees, Francesco Gavioli, Paola Parronchi, Francesco Ciccia, Chiara Cardamone, José Hernández-Rodríguez, Gülen Hatemi, Cemal Bes, Ibrahim A. Almaghlouth, Ángel Robles Marhuenda, Andrés Gonzáles-García, Carla Gaggiano, Amato De Paulis, Mehmet Engin Tezcan, Antonio Luca Brucato, Eugen Feist, Fernando Tornero-Romero, Benoit Suzon, Benson Ogunjimi, Maissa Thabet, Alessandro Conforti, Valentina Pucino, Oksana Boyarchuk, Tetiana Kovalchuk, Marcello Govoni, Annamaria Iagnocco, Maria Sole Chimenti, Abdelhfeez Moshrif, Emanuela Del Giudice, Francesco Carubbi, Şükran Erten, Samar Tharwat, Mohamed Tharwat Hegazy, Ewa Więsik-Szewczyk, Jiram Torres-Ruiz, Eduardo Martín-Nares, Albert Balistreri, Claudia Fabiani, Bruno Frediani, Andrea Hinojosa-Azaola, Özgür Kasapçopur, Luca Cantarini

**Affiliations:** 1 Department of Medical Sciences, Surgery and Neurosciences, Research Center of Systemic Autoinflammatory Diseases and Behçet’s Disease Clinic, University of Siena, Siena, Italy; 2 Azienda Ospedaliero-Universitaria Senese [European Reference Network (ERN) for Rare Immunodeficiency, Autoinflammatory and Autoimmune Diseases (RITA) Center] Siena, Siena, Italy; 3 Department of Precision and Regenerative Medicine and Ionian Area (DiMePRe-J) Policlinic Hospital, University of Bari, Bari, Italy; 4 Gazi University Hospital, Department of Internal Medicine, Division of Rheumatology, Ankara, Türkiye; 5 Internal Medicine Department, Rheumatology and Clinical Immunology Unit, Faculty of Medicine, Cairo University, Giza, Egypt; 6 Joint Academic Rheumatology Program, Medical School, National and Kapodistrian University of Athens, [European Reference Network (ERN) for Rare Immunodeficiency, Autoinflammatory and Autoimmune Diseases (RITA) Center], Athens, Greece; 7 Faculty of Medicine, Università Vita-Salute San Raffaele, Milan, Italy; 8 Unit of Immunology, Rheumatology, Allergy and Rare Diseases, IRCCS Ospedale San Raffaele, Milan, Italy; 9 Division of Pediatric Rheumatology, Department of Pediatrics, Hacettepe University School of Medicine, Ankara, Türkiye; 10 Department of Internal Medicine and Medical Specialties, Rheumatology Unit, AOU Policlinico Umberto I, Sapienza University of Rome, Rome, Italy; 11 Azienda Ospedaliera Universitaria Integrata of Verona, Internal Medicine, Immunology Unit, Verona, Italy; 12 Department of Internal Medicine and Therapeutics, Università di Pavia, Italy; Division of Rheumatology, Fondazione IRCCS Policlinico San Matteo, [European Reference Network (ERN) for Rare Immunodeficiency, Autoinflammatory and Autoimmune Diseases (RITA) Center], Pavia, Italy; 13 Rheumatology and Clinical Immunology, Spedali Civili and Department of Clinical and Experimental Sciences, University of Brescia, [European Reference Network (ERN) for Rare Immunodeficiency, Autoinflammatory and Autoimmune Diseases (RITA) Center], Brescia, Italy; 14 Department of Biotechnological and Applied Clinical Sciences, University of L'Aquila, L'Aquila, Italy; 15 Department of Pediatrics, Pediatric Rheumatology Center (F.L.T.), Giovanni XXIII Pediatric Hospital, University of Bari Aldo Moro, Bari, Italy; 16 Department of Paediatrics, Division of Paediatric Rheumatology, Istanbul University-Cerrahpasa, Faculty of Medicine, Istanbul, Türkiye; 17 University Department of Health Promotion, Mother and Child Care, Internal Medicine and Medical Specialties (PROMISE) “G. D'Alessandro”, University of Palermo, Palermo, Italy; 18 Department of Pediatrics, King Faisal Specialist Hospital and Research Center, College of Medicine, Alfaisal University, Riyadh, Saudi Arabia; 19 Department of Pediatrics, Attikon General Hospital, National and Kapodistrian University of Athens, Athens, Greece; 20 Department of Life Sciences and Public Health, Fondazione Policlinico Universitario A. Gemelli IRCCS, Rome, Italy; 21 Periodic Fever Research Center, Università Cattolica Sacro Cuore, Rome, Italy; 22 Department of Aging, Neurological, Orthopedic and Head and Neck Sciences, Fondazione Policlinico Universitario Agostino Gemelli Istituto di Ricovero e Cura a Carattere Scientifico (IRCCS), Rome, Italy; 23 Department of Health Promotion, Mother and Child Care, Internal Medicine and Medical Specialties, Rheumatology Unit, P. Giaccone University Hospital, University of Palermo, Palermo, Italy; 24 Rheumatology Unit, Department of Medical Sciences and Public Health, University and AOU of Cagliari, Cagliari, Italy; 25 Department of Medical, Surgical and Health Sciences, University of Trieste, Italy, and Clinical Medicine and Rheumatology Unit, Cattinara University Hospital, Trieste, Italy; 26 Centre for Inflammatory Diseases, Department of Medicine, Monash Medical Centre, Monash University, Clayton, VIC, Australia; 27 Pediatric Rheumatology Department of Health Sciences, Magna Graecia University, Catanzaro, Italy; 28 Pediatric Nephrology and Rheumatology Unit, Azienda Ospedaliero Universitaria (AOU) G Martino, Messina, Italy; 29 Rheumatology Unit, Department of Medicine, University of Padua, [European Reference Network (ERN) for Rare Immunodeficiency, Autoinflammatory and Autoimmune Diseases (RITA) Center], Padua, Italy; 30 Rheumatology, Department of Medicine and Surgery, University of Perugia, Perugia, Italy; 31 Clinical and Research Section of Rheumatology and Clinical Immunology, Fondazione Policlinico Campus Bio-Medico, Rome, Italy; 32 Rheumatology and Clinical Immunology, Department of Medicine, University of Rome Campus Bio-Medico, School of Medicine, Rome, Italy; 33 Pediatric Rheumatology Unit, Department of Integrated Maternal-Child and Reproduction Activity, AOU “Policlinico-San Marco”, Catania, Italy; 34 Department of Woman, Child and of General and Specialized Surgery, University of Campania ``Luigi Vanvitelli'', Naples, Italy; 35 Unit of Rheumatology, Department of Medicine, ARNAS Garibaldi Hospital, Catania, Italy; 36 Division of Rheumatology, Department of Internal Medicine, School of Medicine, Marmara University, Istanbul, Türkiye; 37 Department of Rheumatology, “Evangelismos” General Hospital, Athens, Greece; 38 Rheumatic and Systemic Autoimmune Diseases Unit, Department of Interdisciplinary Medicine (DIM), University of Bari Medical School, Bari, Italy; 39 Division of Rheumatology, University of Missouri, Kansas City, MO, United States; 40 Department of Experimental and Clinical Medicine, University of Florence, Florence, Italy; 41 Department of Precision Medicine, Rheumatology Section, University of Campania Luigi Vanvitelli, Naples, Italy; 42 UOC of Internal Medicine, Rheumatology Outpatients Unit, Azienda Ospedaliero-Universitaria San Giovanni di Dio e Ruggi D'Aragona, Salerno, Italy; 43 Department of Autoimmune Diseases, Institut D'Investigacions Biomèdiques August Pi I Sunyer (IDIBAPS), Hospital Clínic of Barcelona [European Reference Network (ERN) for Rare Immunodeficiency, Autoinflammatory and Autoimmune Diseases (RITA) Center], University of Barcelona, Barcelona, Spain; 44 Department of Internal Medicine, Division of Rheumatology, Cerrahpasa Medical School, Istanbul University-Cerrahpasa, Istanbul, Türkiye; 45 Behçet’s Disease Research Center, Istanbul University-Cerrahpasa, Istanbul, Türkiye; 46 Department of Rheumatology, University of Health Sciences Başakşehir Çam and Sakura City Hospital, Istanbul, Türkiye; 47 Rheumatology Unit, Department of Medicine, College of Medicine, King Saud University, Riyadh, Saudi Arabia; 48 College of Medicine Research Center, College of Medicine, King Saud University, Riyadh, Saudi Arabia; 49 Department of Internal Medicine, Autoimmune Disease Unit, Hospital Universitario La Paz, Madrid, Spain; 50 Systemic Autoimmune Diseases Unit, Department of Internal Medicine, Hospital Universitario Ramón y Cajal, IRYCIS, Madrid, Spain; 51 Department of Translational Medical Sciences, Section of Clinical Immunology, University of Naples Federico II, Naples, Italy; 52 Center for Basic and Clinical Immunology Research (CISI), WAO Center of Excellence, University of Naples Federico II, Naples, Italy; 53 Department of Biomedical and Clinical Sciences, Fatebenefratelli Hospital, Università di Milano, Milan, Italy; 54 Department of Rheumatology and Clinical Immunology, Helios Fachklinik Vogelsang-Gommern Klinik für Rheumatologie, Gommern, Germany; 55 Experimental Rheumatology, Otto-von-Guericke Universität Magdeburg, Magdeburg, Germany; 56 Autoimmune Diseases Unit, Department of Internal Medicine, Fundación Jiménez Díaz University Hospital, Madrid, Spain; 57 Department of Internal Medicine, Martinique University Hospital, Fort-de-France, Martinique; 58 Antwerp Center for Translational Immunology and Virology (ACTIV), Center for Health Economics Research and Modeling Infectious Diseases (CHERMID), Vaccine and Infectious Disease Institute (VAXINFECTIO), University of Antwerp, Antwerp, Belgium; 59 Division of Paediatric Rheumatology, Department of Paediatrics, Antwerp University Hospital (UZA), Antwerp, Belgium; 60 Division of Paediatric Rheumatology, Department of Paediatrics, Kidz Health Castle Universitair Ziekenhuis Brussel (UZB), Jette, Belgium; 61 Division of Paediatric Rheumatology, Department of Rheumatology, Ziekenhuis Aan de Stroom (ZAS), Antwerp, Belgium; 62 Internal Medicine Department, Farhat Hached University Hospital, Faculty of Medicine of Sousse, University of Sousse, Sousse, Tunisia; 63 Ospedale San Paolo Di Civitavecchia, U.O. Medicina Generale, ASL Roma 4, Civitavecchia, Italy; 64 Clinical Immunology and Allergy Unit, Department of Clinical and Experimental Medicine, University of Pisa, Pisa, Italy; 65 Department of Children’s Diseases and Pediatric Surgery, I. Horbachevsky Ternopil National Medical University, Ternopil, Ukraine; 66 Rheumatology Unit, Department of Medical Sciences, Azienda Ospedaliero-Universitaria S. Anna-Ferrara, University of Ferrara, Ferrara, Italy; 67 Academic Rheumatology Center, Dipartimento Scienze Cliniche e Biologiche, Università Degli Studi Di Torino, Turin, Italy; 68 Rheumatology, Allergology and Clinical Immunology, Department of Systems Medicine, University of Rome Tor Vergata, Rome, Italy; 69 Rheumatology Department, Faculty of Medicine, Al-Azhar University, Assiut, Egypt; 70 Department of Maternal Infantile and Urological Sciences, Sapienza University of Rome, Polo Pontino, Rome, Italy; 71 Department of Life, Health and Environmental Sciences, University of L'Aquila; Internal Medicine and Nephrology Division, Avezzano-Sulmona-L'Aquila Local Health Authority 1, San Salvatore Hospital, L'Aquila, Italy; 72 Department of Rheumatology, Ankara Bilkent City Hospital, Ankara Yıldırım Beyazıt University, Ankara, Türkiye; 73 Rheumatology and Immunology Unit (S.T.), Internal Medicine Department, Mansoura University, Mansoura, Egypt; 74 Department of Internal Medicine, Faculty of Medicine, Horus University, New Damietta, Egypt; 75 Internal Medicine Department, Rheumatology Unit, Jahra Hospital, Al Jahra, Kuwait; 76 Department of Internal Medicine, Pneumonology, Allergology, Clinical Immunology and Rare Diseases, Military Institute of Medicine, National Research Institute, Warsaw, Poland; 77 Department of Immunology and Rheumatology, Instituto Nacional de Ciencias Médicas y Nutrición Salvador Zubirán, Mexico City, Mexico; 78 Bioengineering and Biomedical Data Science Lab, Department of Medical Biotechnologies, University of Siena, Siena, Italy; 79 Ophthalmology Unit, Department of Medicine, Surgery and Neurosciences, University of Siena, Siena, Italy

**Keywords:** arthritis, autoinflammatory diseases, biologic therapy, personalized medicine, precision medicine

## Abstract

**Objective:**

The primary aim of this study was to assess, in Still’s disease, whether the employment of canakinumab at a strictly on-label dose may increase the likelihood of treatment discontinuation due to study-defined long-term remission (LTR), compared with patients receiving lower doses.

**Methods:**

Patients were drawn from the international Autoinflammatory Disease Alliance (AIDA) Network registry dedicated to Still’s disease and stratified based on the starting canakinumab dose: the on-label group received either 300 mg every 4 weeks or 150 mg every 4 weeks (corresponding to 4 mg/kg), while the underdosed group received 150 mg every 4 weeks (corresponding to a dose not exceeding 3.5 mg/kg). Bayesian regression models were implemented to estimate the probability of achieving long-term remission with subsequent canakinumab withdrawal in the two groups, as well as the mean differences in probabilities and posterior probabilities indicating whether the on-label group was superior in achieving the endpoint.

**Results:**

In total, 131 patients (16.7%) were enrolled, 81 (61.8%) receiving the on-label posology and 50 (38.2%) the underdosed posology. The estimated marginal posterior probability of canakinumab discontinuation due to LTR was 19% (CrI 7.5%–34.6%) in the on-label group and 3.9% (CrI 0.7%–15.2%) in the underdosed group, yielding a mean difference of 15.1% (CrI 1.4%–31.4%) and a posterior probability of 98.4%. This difference remained credible, with posterior probabilities ranging from 97.9% to 99.6%, irrespective of disease course or age at disease onset.

**Conclusion:**

On-label canakinumab dosing appears to increase the likelihood of study-defined LTR with subsequent treatment discontinuation, compared with underdosed treatment strategies.

## Introduction

Still’s disease is a systemic autoinflammatory disorder with a profound impact on patients’ health status and a potential for life-threatening complications. These include macrophage activation syndrome, which represents the most frequent cause of mortality in affected patients, as well as fulminant hepatitis and pulmonary and myocardial involvement (Mitrovic and Fautrel, 2018; Ruscitti et al., 2025a; Ruscitti et al., 2025b). In recent years, several therapeutic options have substantially modified the natural history of the disease, allowing for rapid control of both clinical and laboratory manifestations. In particular, inhibition of interleukin (IL)-1 and IL-6 has proven to be highly effective in these patients, ensuring both efficacy and safety regardless of the timing of treatment initiation relative to disease onset, the pattern of clinical course, which is typically classified as monocyclic, polycyclic, or chronic articular, and across both paediatric and adult populations (Kilic et al., 2025; Kilic Konte et al., 2025; Vitale et al., 2023; Vitale et al., 2022a; Vitale et al., 2020).

Among the questions that remain regarding the treatment of Still’s disease is whether a more aggressive and early biotechnological therapeutic approach can more profoundly impact the pathogenesis of the disease, potentially inducing persistent remission and, ultimately, favouring a monocyclic course. By definition, a monocyclic course is characterized by the complete and definitive resolution of disease within 1 year from onset. This is contrasted with a polycyclic course, in which disease flares recur occasionally and unpredictably, and with a chronic articular course, which involves persistent systemic inflammation that is less intense than in monocyclic or polycyclic forms but predominantly affects the joints (Cush et al., 1987). In this context, canakinumab, one of the two IL-1 inhibitors currently available on the market for the treatment of Still’s disease, is administered at the on-label dose of 4 mg/kg every 4 weeks, up to a maximum of 300 mg every 4 weeks (Ruperto et al., 2012; Ghannam et al., 2021). Since only 150 mg vials are commercially available, adult patients are typically treated with either 150 mg or 300 mg every 4 weeks, depending on how closely this approximates the recommended weight-based dose of 4 mg/kg/4 weeks. However, the use of fixed dosing without adjustment to the 4 mg/kg regimen may result in suboptimal drug exposure: 150 mg every 4 weeks is insufficient for many adults weighing more than 37.5 kg, as well as for numerous adolescent patients whose body weight is comparable to that of adults. Consequently, in such patients, the 4 mg/kg every-4-week regimen is often not strictly followed, and a 150 mg dose corresponds to less than the recommended 4 mg/kg.

The present study aims to investigate whether initiating treatment with the on-label dose can improve outcomes in patients with Still’s disease compared with those receiving a lower dose, particularly in terms of achieving study-defined long-term remission (LTR) sufficient to allow discontinuation of canakinumab.

## Methods

Demographic, clinical, and laboratory data, as well as therapeutic information, were collected from the International AutoInflammatory Disease Alliance (AIDA) registry dedicated to Still’s disease patients (Vitale et al., 2022b). Disease activity at CAN initiation was assessed using the systemic Pouchot score, a validated tool quantifying the burden of systemic manifestations in Still’s disease, calculated by assigning one point to each of twelve disease-related clinical manifestations, with higher scores indicating greater systemic involvement (Pouchot et al., 1991). Patients were included in the study if they met the following criteria: (i) fulfillment of at least one set of classification criteria [for adult patients: Yamaguchi et al., Fautrel et al., and/or Cush et al. criteria (Cush et al., 1987; Yamaguchi et al., 1992; Fautrel et al., 2002); for patients under 16 years of age: the International League of Associations for Rheumatology (ILAR) and/or Pediatric Rheumatology International Trials Organization (PRINTO) criteria (Petty et al., 2004; Martini et al., 2019)]; (ii) a history of treatment with canakinumab; and (iii) administration of canakinumab at specified doses of 150 mg or 300 mg every 4 weeks. Paediatric patients administered with these specified doses were also included in the study.

The primary aim of the study was to assess whether the use of canakinumab at a strictly on-label dose is associated with a higher likelihood of treatment discontinuation due to LTR compared with patients receiving lower doses as early as the initial phase of therapy. For this purpose, patients were stratified based on the dose of canakinumab administered as early as the first 3 months: the on-label group, receiving either 300 mg every 4 weeks or 150 mg every 4 weeks corresponding to 4 mg/kg, and the underdosed group, receiving 150 mg every 4 weeks, corresponding to a dose not exceeding 3.5 mg/kg. The primary endpoint was discontinuation of canakinumab due to achievement of LTR, with remission defined according to the EULAR/PreS recommendations (Fautrel et al., 2024) as complete and sustained clinical inactive disease persisting for at least 6 months. Clinical inactive disease had to be characterized by full resolution of clinical manifestations, including fever, arthritis, and skin rash, along with normalization of laboratory inflammatory markers, namely, erythrocyte sedimentation rate and/or C-reactive protein, resulting in patient-reported wellbeing. Importantly, LTR was not restricted to a fixed duration of 6 months; rather, it referred to a remission period lasting at least 6 months, beyond which the treating physician, in the presence of persistent and complete patient wellbeing, elected to discontinue therapy. The follow-up period extended from symptom onset to the most recent visit recorded in the corresponding AIDA registry (up to December 2025).

Patients included provided their informed consent to participate; the study protocol was conformed to the tenets of the Declaration of Helsinki and was approved by the Ethics Committee of the Azienda Ospedaliero-Universitaria Senese, Siena, Italy in June 2019 (Ref. N.14951).

Statistical analysis was performed using Bayesian regression to evaluate the association between the independent variable, CAN dosing status (on-label *versus* underdosed regimens), and the study endpoint. Regression models were adjusted for covariates, including the baseline glucocorticoids dosage (daily prednisone or equivalent), disease course type (monocyclic, polycyclic or chronic-articular), and age at disease onset, categorized as adult-onset (≥16 years), or childhood onset (<16 years). Models were fitted using the brms package in R Studio (version 4.4.1), which interfaces with Stan for Bayesian inference via Hamiltonian Monte Carlo. The binary outcome, canakinumab discontinuation for LTR, was modeled using a Bernoulli likelihood with a logit link function. Regression coefficients were assigned weakly informative normal priors [Normal(0, 2.5)], while the intercept was assigned a Normal(0, 5) prior. Posterior inference was obtained using Markov Chain Monte Carlo sampling with four chains of 4,000 iterations each. Posterior summaries were reported as means and 95% credible intervals (95% CrIs). To facilitate clinical interpretation, posterior predictive marginal probabilities of achieving the clinical outcome were estimated by fixing the canakinumab dosage independent variable at 1 and 0 while marginalizing the remaining covariates. To assess the specific effects of the adjustment covariates, posterior predictive probabilities conditional on prespecified covariate values were calculated by fixing both the independent variable and all other covariates.

Two sensitivity analyses were performed. First, the Bayesian regression model was repeated using a more conservative prior specification for regression coefficients [Normal(0,2) instead of Normal(0,2.5)] to assess the robustness of the findings to prior assumptions in the context of a limited number of outcome events. Second, a Bayesian Cox proportional hazards model was fitted to account for differences in follow-up duration, censoring, and time at risk, using treatment discontinuation due to long-term remission as the event of interest and censoring patients at their last available follow-up visit.

Associations were considered statistically significant if the 95% CrI excluded the null value (OR = 1) and/or if the posterior probability exceeded 97.5%, whereas posterior probabilities above 95% were considered indicative of a trend toward significance. Descriptive statistics, including mean, median, standard deviation (SD), interquartile range (IQR), frequency counts and corresponding percentages, were also reported.

## Results

**FIGURE 1:**
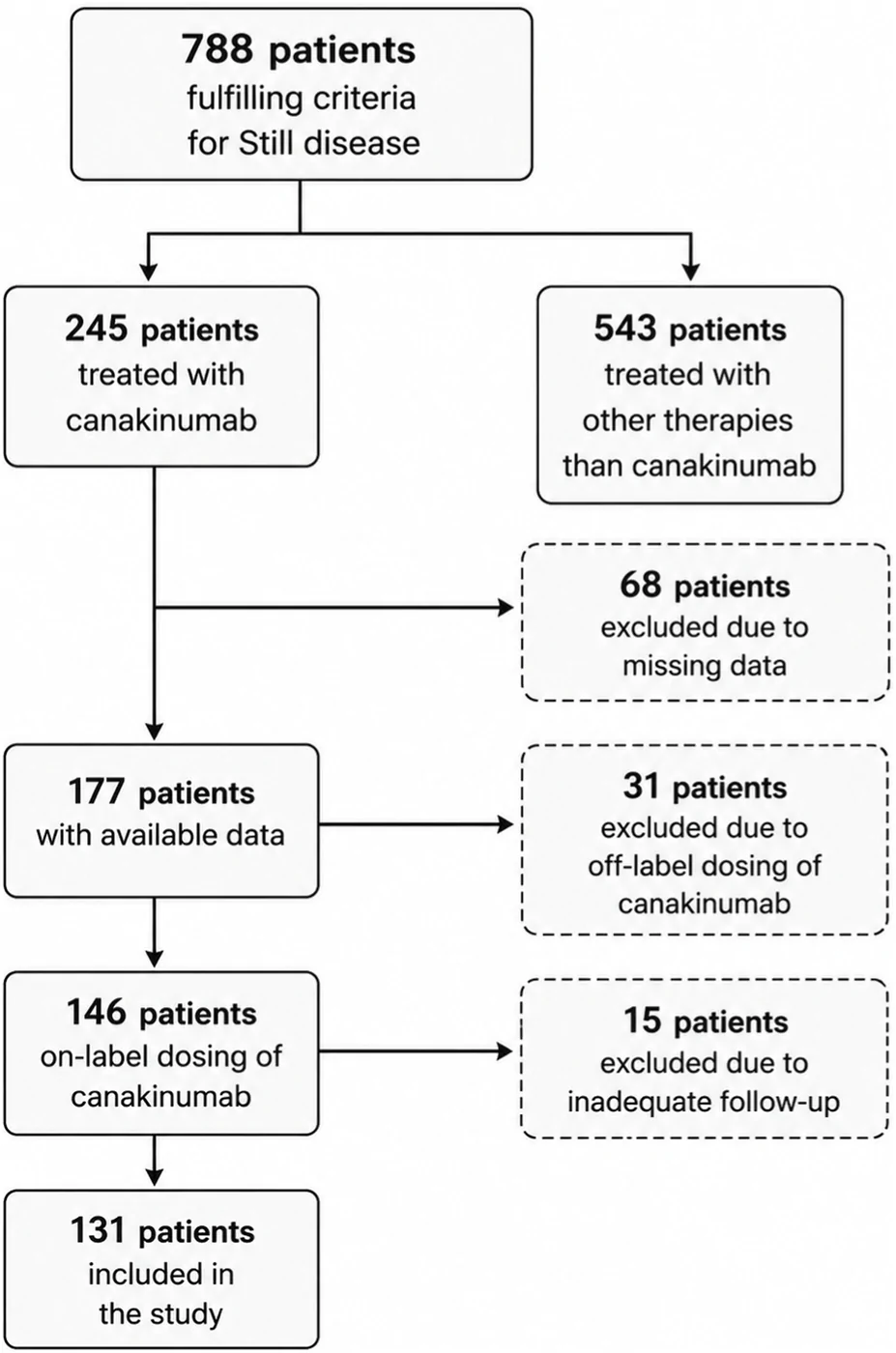
Flowchart of patient’s selection. Among 788 patients fulfilling classification criteria for Still’s disease and enrolled in the AIDA Network Registry, 245 had received canakinumab and were assessed for eligibility. After exclusion of patients with missing data (n = 68) and those receiving off-label canakinumab dosing regimens (n = 31), 146 patients remained eligible. A further 15 patients were excluded because of inadequate follow-up, resulting in a final study population of 131 patients included in the analysis.

As a whole, 788 individuals were enrolled in the AIDA registry dedicated to Still’s disease, with 131 (16.7%) meeting the study inclusion criteria. Figure 1 provides the flow chart describing how patients were selected for the inclusion in the study. Eighty-one patients (61.8%) received the on-label posology as early as the first 3 months of canakinumab administration, whereas 50 patients (38.2%) were treated with the underdosed posology. Twenty-two (16.8%) patients were younger than 16 years, of whom 13 (9.9%) received canakinumab at underdosed posology and 9 (6.9%) received the on-label posology. Ninety out of 131 patients (68.7%) had received at least one biotechnological therapy before CAN initiation. Information on these treatments was available for 73/90 patients. In particular, 30/73 patients were treated with CAN underdosed and 43/73 with CAN on-label, with non-statistically significant difference between groups, as described in Table 1. Anakinra was the most frequently administered biotechnological agent prior to CAN, having been used in 24/30 patients (80.0%) in the underdosed group and 39/43 patients (90.7%) in the on-label group. Previous exposure to anti-IL-6 therapies was reported in 14/30 (46.7%) and 9/43 (20.9%) patients, respectively, whereas prior anti-tumor necrosis factor treatment was observed in 4/30 (13.3%) and 5/43 (11.6%) patients, respectively. Demographic, clinical and therapeutic data from the cohort are shown in Table 1.

**TABLE 1:** Demographic characteristics and disease-related parameters of patients treated with underdosed canakinumab posology (group A) and on label canakinumab posology (group B). Acronyms: cDMARDs, conventional disease modifying anti-rheumatic drugs; IQR, interquartile range; LTR, long term remission; SD, standard deviation.

Variable	Underdosed posology (group A), n = 50	On label posology (group B), n = 81	Δ_B-A_ (95% CrI of Δ_B-A_)	P (A > B)	P(B > a)
Age at disease onset in years, mean ± SD	28.1 ± 18.1	35.9 ± 16.1	7.76 (1.79–13.93)	0.7%	99.3%
Age at the start of canakinumab in years, mean ± SD	30.9 ± 18.1	37.5 ± 14.9	6.46 (0.01–12.7)	2.5%	97.5%
Disease duration at the start of canakinumab in months, median (IQR)	18.5 (54)	16 (44)	−2.73 (−24.00 to 13.01)	60.2%	36.6%
Treatment duration in months, median (IQR)	24 (39)	23 (31.5)	−4.5 (−21 to 8)	61%	25%
Systemic score at the start of canakinumab, median (IQR)	1 (3)	2 (4)	0.485 (−1–2)	18.6%	55.1%
Dosage of daily oral steroid at the start of canakinumab, median (IQR)§	9.65 (17.3)	20 (40.6)	9.23 (0.00–19.25)	2.4%	95.9%
Concomitant cDMARD at the start of canakinumab*	12 (24%)	17 (21%)	−3% (−18%–12%)	64.8%	35.2%
Concomitant cDMARD used while on canakinumab	15 (30%)	21 (25.9%)	−4% (−20%–0.12%)	69%	31%
Previous biotechnological therapy	30 (60%)	43 (53.1%)	7.5% (−5.7% to 22%)	12.3%	87.7%
Adult onset	37 (74%)	72 (88.9%)	15% (1.5%–29.4%)	1.5%	98.5%
Pediatric onset	13 (26%)	9 (11.1%)	−14.8% (−29.4% to −1.2%)	98.2%	1.9%
Body weight, kg, mean ± SD	61.7 ± 13.0	76.8 ± 20.3	15.18 (8.5–22)	0%	100%
Disease course#	​	​	​	​	​
Monocyclic disease course	7 (16.3%)	10 (16.4%)	−1.7% (−14.5%–14.3%)	48%	52%
Polycyclic disease course	20 (46.5%)	33 (54.1%)	7.6% (−12.2%–26.7%)	21.5%	78.5%
Chronic-articular disease course	16 (37.2%)	18 (29.5%)	−7.8% (−26.6%–10.2%)	79.7%	20.3%
Canakinumab discontinuation for LTR	2 (4%)	7 (8.6%)	4.5% (−4% to −12.3%)	13.2%	86.8%
Canakinumab discontinuation for effectiveness issues	7 (14%)	5 (6.2%)	−7.8% (−2%–2.6%)	92.3%	7.7%
Canakinumab dosage decrease during follow-up	15 (30%)	13 (16%)	−14.1% (−29.4%–0.8%)	96.7%	3.3%

Δ_B-A_: posterior mean difference in the mean/median/proportion between groups B and A estimated using a Bayesian model.

P (A > B): posterior probability that the mean/median/proportion is higher in group A than in group B.

P (B > A): posterior probability that the mean/median/proportion is higher in group B than in group A.

§ Glucocorticoids dosage as prednisone or equivalent; *Within the first 3 months; #Already provided in 43/50 (86%) patients in group A and 61/81 (75.3%) cases in group B.

In total, 9 (6.9%) patients discontinued due to LTR after a median treatment duration of 24 (IQR 9.5, range 15–38) months. The median follow-up after canakinumab discontinuation was 7 (IQR 7, range 3–38) months. Beyond the patients who discontinued, a decrease in canakinumab dosage was observed over time in 28 cases (21.4%) during a phase following the first 3 months of therapy. An increase in canakinumab dosage was performed in 7 (5.3%) patients; none of them were found to experience treatment discontinuation over time.

Based on Bayesian regression analysis, the estimated marginal posterior probability of canakinumab discontinuation due to LTR was 19% (CrI 7.5%–34.6%) in patients treated with the on-label posology, compared with 3.9% (CrI 0.7%–15.2%) in those receiving the underdosed posology. The posterior difference in probabilities of achieving the primary outcome between the two dosing strategies was 15.1% (CrI 1.4%–31.4%), with a posterior probability of 98.4% favouring the on-label regimen for treatment discontinuation due to LTR.

As a sensitivity analysis, a Bayesian Cox proportional hazards model accounting for differences in follow-up duration was performed. On-label CAN dosing remained strongly associated with treatment discontinuation due to LTR (β = 18.67, 95% CrI 1.50–83.66), with a posterior probability exceeding 99% that the effect was in favour of the on-label regimen.

A second sensitivity analysis conducted to assess the robustness of the results to more conservative prior assumptions yielded results consistent with those of the primary model. In particular, the association between on-label CAN dosing and LTR-based treatment discontinuation remained consistent in direction, with only limited attenuation of the estimated effect size (OR 9.75, 95% CrI 0.91–162.59), and a posterior probability of 97.1% favouring the on-label regimen.

To assess whether the duration of Still’s disease at the start of CAN influenced the probability of achieving treatment discontinuation due to LTR, patients were stratified according to the time elapsed between disease onset and initiation of treatment. Table 2 shows the results of the Bayesian regression for each stratum. Across all strata, the on-label canakinumab dosage was associated with at least a 97.9% posterior probability of favouring treatment discontinuation due to LTR compared with the lower dose, regardless of disease duration at treatment initiation.

**TABLE 2:** Probability of achieving long-term remission (LTR) leading to canakinumab discontinuation according to the initial treatment dose (on-label regimen versus underdosed regimen), stratified by disease duration at treatment initiation, age at disease onset (adults if ≥ 16 years, otherwise pediatric), and disease course. P(LTR | On-label regimen) and P(LTR | Underdosed regimen) represent the probability of achieving LTR given the on-label and underdosed canakinumab dosing regimens, respectively. ΔP denotes the absolute difference in the probability of achieving LTR and subsequent treatment discontinuation between the two dosing regimens, along with the corresponding 95% credible interval (CrI) and the posterior probability that ΔP > 0, i.e., the probability that the difference between the on-label and underdosed regimen is greater than zero.

Disease duration at the start of canakinumab	P(LTR|On-label regimen)	P(LTR|Underdosed regimen)	Δp	95% CrI of ΔP	P(ΔP) > 0
1 month	18.9%	3.7%	15.2%	0.5%–33.9%	97.9%
6 months	18.8%	3.7%	15.1%	0.5%–33.3%	97.9%
12 months	18.5%	3.6%	14.9%	0.5%–32.7%	97.9%
24 months	18.1%	3.5%	14.6%	0.6%–31.3%	97.9%
Age at disease onset					
Adults	19.1%	2.6%	16.9%	3.9%–33.8%	99.5%
Children	32.2%	6%	26.2%	2.0%–61.6%	99.5%
Disease course					
Monocyclic	32%	4.9%	27.1%	5.9%–55.6%	99.6%
Polycyclic	13.8%	1.7%	12.1%	0.8%–32.7%	99.6%
Chronic articular	19.7%	2.8%	16.9%	1.7%–42.7%	99.6%

Based on these results, we evaluated whether the study hypothesis was valid in both children and adults. In both cases, administering the on-label canakinumab dosage was associated with higher probability to induce LTR with subsequent treatment discontinuation with a posterior probability of 99.5% in each group, as shown in Table 2. Similarly, when considering the type of disease course, the study hypothesis was confirmed, with a posterior probability of 99.6% in patients with monocyclic, polycyclic, and chronic articular disease. Table 2 provides details for each type of disease course.

**FIGURE 2:**
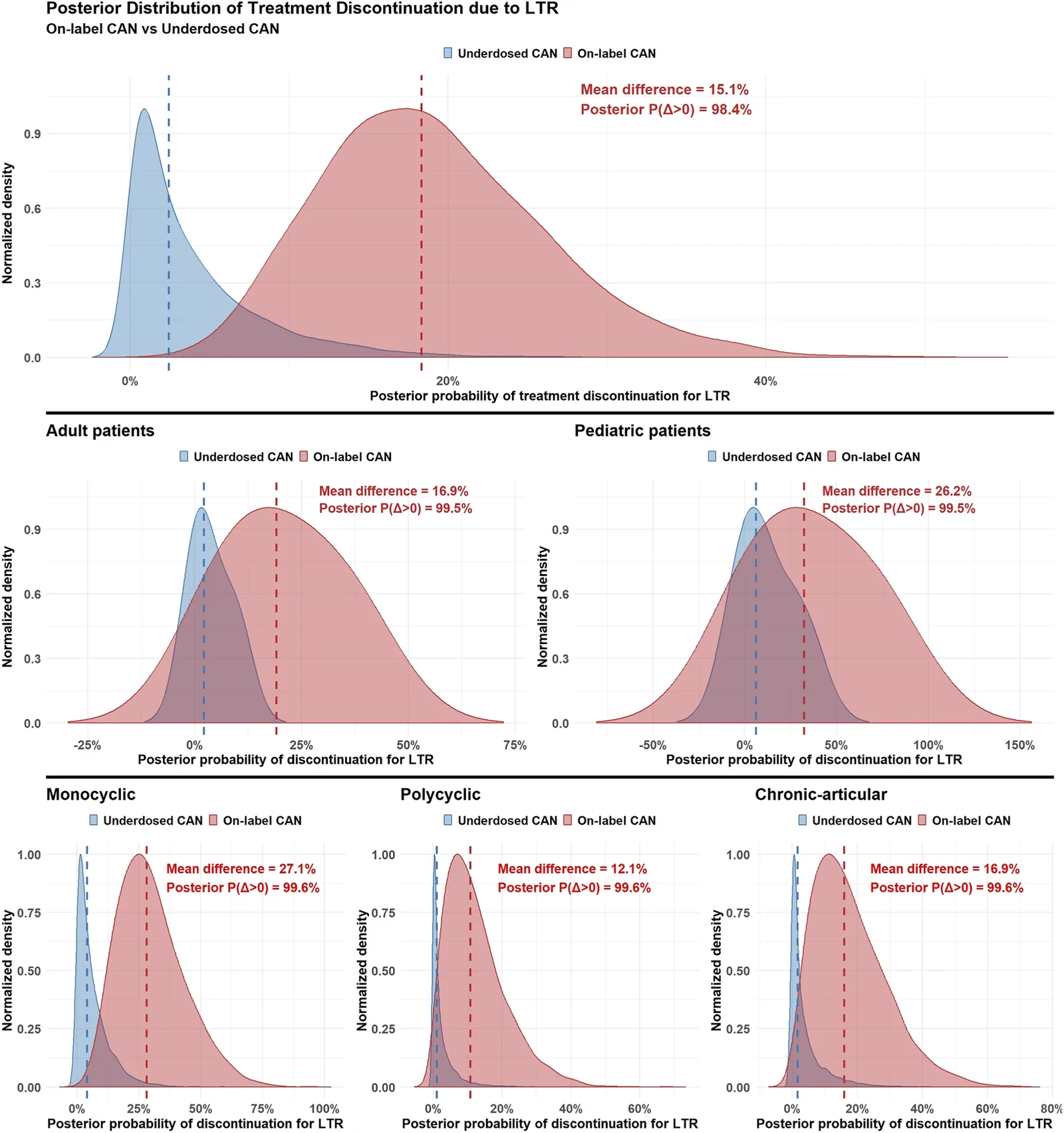
Analysis of posterior distributions for the probability of canakinumab (CAN) withdrawal for long-term remission (LTR). Shaded areas represent the probability density for on-label CAN dosages (red) and underdosed canakinumab dosage (blue), with dashed lines indicating the means of the distributions. The upper panel shows the analysis in the overall population, adjusted for age at onset and disease course type. The middle panel distinguishes the population by pediatric (<16 years) versus adult (≥16 years) onset, while still adjusting for disease course type. The lower panel displays the population stratified by disease course (monocyclic, polycyclic, and chronic-articular), adjusted for age at onset. In all models, the posterior probability of superiority for on-label CAN dosing (Δ > 0) exceeds 97.5%, which is considered statistically significant in this study. The displayed distributions represent posterior density estimates. Apparent extensions beyond the theoretical probability range result from density smoothing procedures and should not be interpreted as observed probabilities below 0% or above 100%. Abbreviation: CAN, canakinumab.

Figure 2 graphically depicts the posterior probability distributions for achieving long-term remission with subsequent canakinumab withdrawal, both in the overall population and stratified by age at onset and disease course type.

## Discussion

The inhibition of IL-1 has represented a major advance in the management of Still’s disease, providing effective disease control in most patients, both clinically and biochemically (Vitale et al., 2023; Vitale et al., 2022a; Fautrel et al., 2024). Nevertheless, current clinical research should now focus on elucidating strategies to achieve sustained LTR and, potentially, definitive disease resolution. In this context, the present study aimed to determine whether initiating treatment at a dose lower than that recommended in the prescribing information could negatively impact the likelihood of achieving LTR and the subsequent discontinuation of therapy. Indeed, the study results suggest that targeting the disease with an adequate dosage may promote LTR-induced treatment discontinuation. Specifically, the posterior probability of discontinuing canakinumab due to LTR was 15.1% higher in patients treated with the on-label regimen compared with those receiving an underdosed regimen, a difference supported by a high posterior probability of 98.4%. This finding held true when stratifying the sample by both adult and pediatric patients and by type of clinical disease course. Interestingly, this effect does not seem to be influenced by a specific therapeutic “window of opportunity”, as it was observed regardless of the time elapsed between symptom onset and the initiation of canakinumab.

It is noteworthy that LTR leading to canakinumab discontinuation is generally more likely when using the on-label dose in both adult and pediatric patients; however, this effect appears particularly pronounced in pediatric patients. Indeed, among pediatric patients treated with the on-label regimen, the probability of achieving LTR increased sharply to 32.2%. Although the number of pediatric patients was small, as they are generally treated using weight-based dosing rather than fixed 150 mg or 300 mg vials, these data suggest that the likelihood of achieving LTR may be even more pronounced in pediatric patients and that this propensity could be leveraged by using an adequate dosage.

Although the use of the appropriate canakinumab dose appears to play a significant role in inducing LTR and enabling subsequent treatment discontinuation regardless of disease course, this effect appears to be particularly pronounced in patients with monocyclic disease. In this subgroup, the difference in the probability of achieving LTR between the on-label regimen and the underdosed posology is approximately 27%, compared to 12%–16% in other types of disease courses. This may indicate that early administration of an adequate canakinumab dose could specifically promote a monocyclic disease pattern, as the monocyclic course might result from more intensive IL-1 inhibition, which in turn allowed subsequent treatment discontinuation due to LTR. Indeed, interpreting the data in reverse, that is assuming patients were classified as monocyclic simply because they were able to discontinue therapy due to LTR, does not explain why this effect would be more likely in patients treated with the on-label regimen rather than the underdosed approach.

As shown in Table 1, several differences emerged between patients treated with canakinumab at the on-label dose compared with those receiving a lower dose. However, most of these differences appear to reflect the intrinsic demographic and clinical characteristics of the study population rather than treatment-related imbalances. Although previous exposure to anti-IL-6 therapy was numerically more frequent in the underdosed group, this difference did not reach statistical significance. Moreover, previous anti-IL-6 therapy should not necessarily be regarded as a marker of greater disease refractoriness, as the choice of biologic therapy in Still’s disease may also be influenced by disease phenotype and local treatment strategies across participating centers. Therefore, this finding should be interpreted within the overall clinical context rather than as evidence of differences in disease severity between the two groups. Specifically, age at disease onset and, consequently, age at canakinumab initiation were significantly higher in patients treated with the on-label regimen. Indeed, this group included patients receiving 300 mg every 4 weeks, who were more likely to be adults. Consistent with this, adult-onset Still’s disease was more prevalent in the on-label group.

Baseline corticosteroid dose, expressed as mg/day of prednisone equivalent, tended to be higher in the on-label group. As previous evidence suggests that higher corticosteroid exposure at baseline may be associated with a monocyclic disease course in Still’s disease (Ruscitti et al., 2019), all results reported in this study were adjusted for the daily glucocorticoid dose administered at treatment initiation.

There was a trend toward significance regarding the higher frequency of canakinumab dose reductions during follow-up, favoring the underdosed group. Notably, median treatment duration was similar between groups and tended to be longer in the underdosed cohort. These findings indicate that, while dose reduction was the primary mode of treatment adjustment in the underdosed group, patients in the on-label group were more often able to discontinue therapy entirely, with dose reductions occurring less frequently.

This study has several limitations. In particular, besides the limited number of patients, which is still considerable given the rarity of the disease and the currently available data on canakinumab use in Still’s disease, the study is observational in nature, and follow-up after treatment discontinuation remains short. However, given that only nine patients achieved the primary endpoint, the precise magnitude of the estimated treatment effect should be interpreted with caution. Nevertheless, sensitivity analyses using alternative prior specifications and a Bayesian time-to-event approach yielded consistent results, with the direction of the association between on-label CAN dosing and treatment discontinuation due to LTR remaining unchanged across all analyses. These findings support the robustness of the qualitative conclusions despite the limited number of events. Furthermore, as with all registry-based studies, the observational nature of the study may have introduced variability in clinical management across participating centers, including differences in CAN withdrawal strategies and timing. In addition, the possibility of residual confounding cannot be completely ruled out, despite adjustment for clinically relevant baseline characteristics. Unmeasured factors influencing treatment decisions, disease severity, or physician prescribing behavior may have contributed to the observed differences between groups. Also, although patients fulfilled the predefined criteria for LTR, the median post-discontinuation follow-up was 7 months. Therefore, the durability of remission beyond the available observation period cannot be firmly established, and studies with longer post-discontinuation follow-up are needed to confirm the persistence of remission after treatment withdrawal. In addition, due to the lack of established guidelines on how and when to discontinue canakinumab, it was not possible to provide a uniform definition of LTR, which was left to the clinical judgment and individual assessment of each treating physician. In the future, with more widespread use of canakinumab and after a sufficiently long follow-up, it will be possible to conduct studies that confirm the observations reported in this study. Nevertheless, using a Bayesian statistical approach, which is preferred over frequentist methods to address the issue of small sample sizes, this work suggests that initiating canakinumab at an adequate dose favours the achievement of LTR sufficient to allow treatment discontinuation. This has important implications for patients in terms of quality of life and the ability to discontinue therapy upon achieving remission. From a pharmacoeconomic perspective, investing more upfront may increase the likelihood of long-term savings, as it enhances the probability of treatment discontinuation over time. Therefore, opting for a reduced canakinumab dose of 150 mg every 4 weeks instead of 300 mg, even if this prevents achieving the recommended 4 mg/kg per 4 weeks, may decrease the likelihood of treatment discontinuation due to LTR and, over the long term, could lead to higher overall healthcare costs.

In conclusion, the use of on-label canakinumab dosage appears to enhance the likelihood of study-defined LTR with subsequent treatment discontinuation. While these findings require confirmation in larger cohorts, they underscore the potential benefit of optimized dosing to maximize IL-1 inhibition, thereby facilitating LTR and therapy cessation.

## Data Availability

The raw data supporting the conclusions of this article will be made available by the authors, without undue reservation.
